# Extracellular vesicles released from p18 overexpressing pulmonary endothelial cells are barrier protective – potential implications for acute respiratory distress syndrome

**DOI:** 10.1177/2045894020951759

**Published:** 2020-09-21

**Authors:** Elizabeth O. Harrington, Julie Braza, Aparna Shil, Havovi Chichger

**Affiliations:** 1 Vascular Research Laboratory, Providence Veterans Affairs Medical Center, Providence, RI, USA; 2 Department of Medicine, Alpert Medical School of Brown University, Providence, RI, USA; 3 School of Life Sciences, Anglia Ruskin University, Cambridge, UK

**Keywords:** basic science research, cell biology, pulmonary biology, endothelium, vascular biology

## Abstract

The novel endosome protein, p18, and the early endosome GTPase, Rab4, play a significant role in protecting the pulmonary vasculature against permeability associated with acute respiratory distress syndrome. Recently, endothelial-derived extracellular vesicles have been identified to play a key role in the endothelial permeability associated with acute respiratory distress syndrome. Therefore, we investigated the effect of these microparticles, released from endothelial cells overexpressing p18 and Rab4, on pulmonary endothelial barrier function. Endothelial-derived extracellular vesicles isolated from lung microvascular endothelial cells which overexpressed cDNA for wild-type p18 protected a naïve monolayer against lipopolysaccharide-induced permeability. In contrast, endothelial-derived extracellular vesicles from cells overexpressing the non-endosomal binding p18 mutant (p18^N39^) exerted no protective effect on the endothelial monolayer. Cells overexpressing either dominant active or inactive Rab4 released endothelial-derived extracellular vesicles which had no effect on lipopolysaccharide-induced permeability. miRNA analysis and permeability studies of endothelial-derived extracellular vesicle isolated from wild-type p18-overexpressing cells demonstrates that let-7i-5p, miR-96-5p, and miR-137-3p are endothelial-derived extracellular vesicle cargo which exert protective effects on the pulmonary endothelium. Finally, we observed down-regulation of p18 protein expression in both the lung and endothelium in an in vivo and in vitro model of acute respiratory distress syndrome. These results demonstrate that endothelial-derived extracellular vesicle released from cells overexpressing p18, but not Rab4, contain miRNA cargo which likely promote a barrier-protective effect on the pulmonary endothelium in settings of acute respiratory distress syndrome. Findings indicate the importance of p18 in the pulmonary vasculature and demonstrate that targeting this protein may provide a novel therapeutic strategy to reduce endothelial permeability associated with acute respiratory distress syndrome.

## Introduction

Over 10% of patients in intensive care units worldwide suffer from acute respiratory distress syndrome (ARDS), associated with a mortality rate of nearly 40%.^1,2^ The main predisposing factors which lead to ARDS are pneumonia, major surgery, trauma, or sepsis.^3^ Patients with ARDS suffer from acute hypoxemic respiratory failure associated with low oxygen partial pressure (<300 mmHg) despite high positive end-expiratory pressure (>5 cmH_2_O).^3^ Due to the complexity and diversity of patient cases, mechanical ventilation is one of the key therapeutic approaches for ARDS to improve hypoxemia; however, this treatment has been linked to worsening of the respiratory failure for some patients.^4^ One of the hallmarks of ARDS is an increase in lung endothelial permeability, associated with the development of pulmonary edema in these patients.^3^ It is therefore vital to understand the mechanisms which regulate endothelial permeability, with the aim of reducing respiratory failure in patients with ARDS.

Endothelial-derived extracellular vesicles (EDEVs) have been identified to play a role in ARDS as a mechanism of communicating pathological signals and as potential biomarkers for the disease.^5,6^ These vesicles range in size from 50 nm up to 1 µm and are shed from activated endothelial cells in response to stimuli such as cytokines, mechanical stress, or the endotoxin lipopolysaccharide (LPS).^7,8^ The cargo of EDEVs, surrounded by an intact lipid bilayer, includes a sampling of the endothelial parent cell contents, such as surface proteins, lipids, miRNAs, or transcription factors or organelles.^9,10^ In various models of ARDS, an increase in EDEVs in the circulation has been demonstrated with downstream effects on the vasculature through reduced nitric oxide production, coagulation, elevated release of inflammatory cytokines, and disruption of the endothelial barrier.^6,10,11^ Studies into the composition of these pathogenic EDEVs demonstrate a role for structural and surface proteins, such as vinculin and moesin, which have been linked to increased permeability in the pulmonary endothelium.^8,12,13^ In recent years, miRNA have been identified as extracellular vesicle cargo which are altered dramatically in disease states such as hyperoxia and inflammation.^14,15^ These small non-coding RNA molecules target the majority of protein-coding transcripts and, as such, are involved in nearly all pathological processes.^16^ Understanding the miRNA composition and release of EDEVs therefore presents a potential therapeutic approach to reduce vascular leak, and therefore pulmonary edema, in settings of ARDS.

We have previously demonstrated a role for two endosomal proteins, Rab4 and p18, in reducing pulmonary endothelial permeability in both in vitro and in vivo models of ARDS.^17,18^ Both Rab4 and p18 bind to the early endosome to facilitate trafficking of vascular endothelial (VE)-cadherin to the endothelial cell surface, to promote formation of the adherens junction, and tighten the endothelial barrier. Rab4 is a GTPase which binds to the early endosome to regulate rapid shuttling of cargo to the cell surface for exocytosis.^19^ In contrast, p18 has been localized to the early and late endosome where it plays a role in trafficking and mTOR signaling, respectively.^17,20,21^ Given the protective effect of these two endosomal proteins on the pulmonary vasculature, and the emerging role of EDEVs in regulating the endothelial barrier, we sought to establish whether release of these microparticles, from cells overexpressing Rab4 and p18, are linked to barrier function in the pulmonary endothelium.

## Materials and methods

### Cell lines and reagents

All materials were obtained from Sigma-Aldrich (St. Louis, MO) unless otherwise stated. Rat lung microvascular endothelial cells (LMVEC; VEC Technologies, Rensselaer, NY) were maintained in MCDB-131 (VEC Technologies) and used between *passages 3* and *11*. LPS (endotoxin) from *Escherichia coli* serotype 0111:B4 was obtained from Enzo Life Sciences (Plymouth Meeting, PA). *Pseudomonas aeruginosa* strain PA103 was a kind gift from Dr. Troy Stevens (University of South Alabama, Mobile, AL). The vector encoding wild-type p18 (GFP-p18^wt^) and mutant p18, lacking endosome binding region (p18^N39^) were a kind gift from Shigeyuki Nada^20^ (Department of Oncogene Research, Osaka University). p18 siRNA (SR513910) and the non-specific, scrambled control siRNA were purchased from Origene (Herford, Germany). The vectors encoding dominant active (Rab4^Q67L^) and dominant negative (Rab4^S22N^) Rab4 were a kind gift from Mary McCaffrey (Cell and Molecular Biology, University College Cork).^22^ Green fluorescent protein vector (pGFP-C1) was obtained from Clontech (Mountain View, CA). Polyjet was obtained from SignaGen Laboratories (Rockville, MD), and antibodies directed against p18 were obtained from Abcam (Cambridge, MA). miRNA mimics and DharmaFECT™ reagent 4 were obtained from Dharmacon (Cambridge, UK): rno-miR-30a-5p (C-320328-03), rno-let-7i-5p (C-320293-05), rno-miR-103-3p (C-320345-03), rno-miR-96-5p (C-320339-03), rno-miR-196a-5p (C-320241-03), rno-miR-324-5p (C-320341-03), and rno-miR-137-3p (C-320368).

### In vivo model of ARDS

A single dose of live bacteria (*P. aeruginosa PA103*), or vehicle of phosphate buffered saline (PBS), was administered to a total of 10 male C57BL6 mice via intratracheal injection (10^7^ CFU). At four hours following *PA103* delivery, mice were delivered a sub-lethal dose of pentobarbitone and anesthetic effects were tested by toe pinch. Lungs were removed from mice, prior to exsanguination under anesthetic, snap frozen, and stored at –80℃ until use. All animal experimental protocols were approved by the Institutional Animal Care and Use Committees of the Providence Veterans Affairs Medical Center and our University, and comply with the Health Research Extension Act and U.S. Public Health Service policy.

### Western blotting

Lungs were homogenized for two minutes in homogenization buffer (20 mM 4-(2-hydroxyethyl)piperazine-1-ethanesulfonic acid (HEPES) pH 7.9, 1.5 mM NaCl, 0.25 M sucrose, 0.2 mM EDTA, 200 mM phenylmethylsulfonylfluoride (PMSF), 0.5 mM 1,4-dithiothreitol (DTT), and 1.5 mM MgCl_2_). Protein concentration was then analyzed and 100 µg protein was prepared in Laemmli buffer for Western blot analysis. Western blotting was performed on lung homogenates or cell lysates, as previously described.^17^ Previously verified antibodies directed against p18 (Abcam, ab121157) were used, with actin (Santa Cruz, C-11) as a load control.^17^ Densitometry of Western blotting data was quantified using ImageJ and normalized to controls.

### Extracellular vesicle isolation

Rat LMVECs were transiently transfected, using Polyjet reagent, with plasmid cDNA encoding GFP-tagged constructs: wild-type (p18^wt^) and non-endosomal binding (p18^N39^) p18, dominant active (Rab4^Q67L^), and non-endosomal binding (Rab4^S22N^) Rab4, or GFP vector control. Alternatively LMVECs were transiently transfected with p18 or scrambled control siRNA (300 nM) using DharmaFect4. Overexpression of constructs was assessed by GFP fluorescence quantified using the Victor fluorometer (Perkin Elmer). At 42 h post-transfection, cells were exposed to LPS (1 µg/ml) for a further six hours. Media from cells was then removed and centrifuged at 300 × *g*, 4℃ for 10 min twice, with pellet discarded each time. Supernatant was then centrifuged at 100,000 × *g*, 4℃ for one hour. The pellet, comprising of EDEVs, was resuspended in sterile Dulbecco's PBS (DPBS) for permeability assays and quantification using qNano Gold (Izon Science) at 0.5 V, 45 mm stretch, 15 mbar pressure, using the NP200 pore and normalized to media as a control, RNAlater for miRNA analysis, or lysed for protein concentration analysis.^23,24^

### Permeability measurements

Changes in monolayer resistance were measured using the electrical cell impedance sensor (ECIS) technique and fluorescein isothiocyanate (FITC) dextran permeability technique^25^ as previously described.^17^ Naïve, un-transfected LMVECs were plated on collagen-coated ECIS arrays or Transwell inserts and cultured for 24 h, prior to exposure to freshly-prepared EDEVs, in the presence and absence of LPS (1 µg/ml). Permeability was measured for 10 h (ECIS) or at 10 h (FITC dextran monolayer flux). The latter studies were performed using 10 µg/ml FITC-conjugated 4 kD dextran which was added to the upper chamber for 30 min prior to the collection period. Collected media (100 µl) from the lower chamber was measured for FITC concentration using fluorometry (495/520 nm ex/em). Permeability (%) was calculated by fluorescence accumulated in the lower chamber divided by fluorescence in the upper chamber, multiplied by 100 and data was normalized to GFP vehicle.

### miRNA analysis

miRNA cargo in isolated EDEVs was assessed using the miScript miRNA PCR array Rat miFinder kit (Qiagen MIRN-001Z). Manufacturer’s instructions were followed for the isolation of RNA from isolated EDEVs and the array was run as indicated in guidelines. Data were collected using the Roche Light cycler 480. The second derivative maximum setting was used to obtain C_T_ values from the Light Cycler. An automated baseline was established using the Light Cycler, and the threshold value for all profiler arrays was established within the lower third of the linear phase of amplification plots. Raw C_T_ data were then exported to Excel for further analysis. Data were normalized for miRNA expression to stably-expressed housekeeping genes (*SNORD68, SNORD95*, and *SNORD96A*).

### miRNA mimic transfection

miRNA mimics, scrambled non-specific (ns) miRNA, and cel-miR67-Dy547 control (10 nM) were transiently transfected into LMVEC using the DharmaFECT™ 4 reagent protocol, as per manufacturer’s guidelines, for 48 h. Transfection efficiency was established using cel-miR67-Dy547 control assessed with fluorometry (557/570 nm ex/em).^26^ Transfected LMVECs were plated on transwell inserts and monolayer permeability was studied as described in “*permeability measurements*”. Cell viability of LMVEC transfected with miRNA mimics was assessed using CCK8 assay, as per the manufacturer’s guidelines, with absorbance read at 450 nm.^25^

### Statistical analysis

The experimental number is presented in the legend for each experiment. For two groups, the variance in data sets was analyzed using the Mann–Whitney test followed by the T-test. In the case of miRNA analysis, the Benjamini–Hochberg correction was used with the R “stats” package. For three or more groups, variance was assessed by using Bartlett’s test with data sets not reaching significance studied by Kruskal–Wallis test followed by Dunn’s test. Significance was reached when *p* < 0.05. Values are presented as mean ± standard error mean (S.E.M.).

## Results

### Endothelial cells overexpressing endosomal p18 release EDEVs which are protective against LPS-induced barrier permeability

**Fig. 1.**
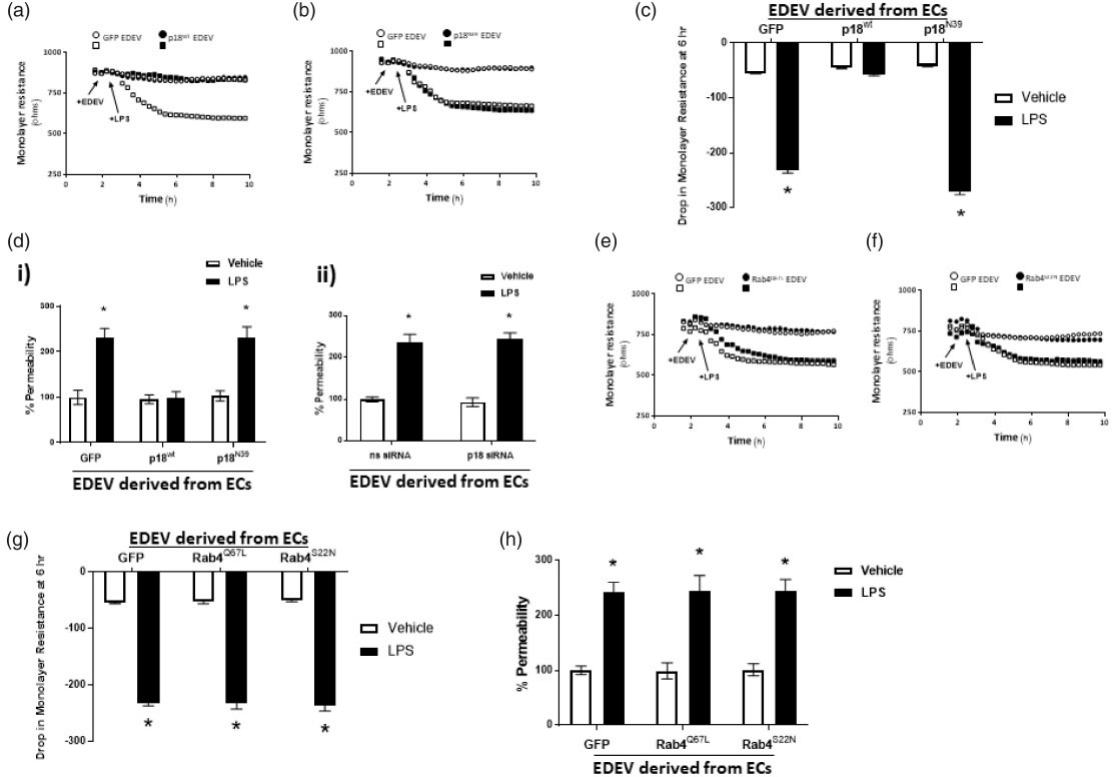
LPS-induced permeability is attenuated by extracellular vesicles isolated from endothelial cells overexpressing wild-type p18, but not dominant positive Rab4 (Rab4^S22N^) or non-endosomal p18 and Rab4 mutants (p18^N39^ and Rab4^Q67L^). Equivalent numbers of LMVECs were transiently transfected with p18^wt^ (*panel a, c, d(i)*) p18^N39^ (*panel b, c, d(i)),* Rab4^Q67L^ (*panel e, g, h*), Rab4^S22N^ (*panel f, g, h)*, and GFP cDNA as a vector control. Alternatively knockdown of p18 expression was performed in LMVEC with non-specific siRNA used as a control (*panel d(ii)).* Following 48 h, extracellular vesicles were isolated from the endothelial monolayer and assayed for concentration and protein concentration. Naive, untransfected LMVEC monolayers were (all panels) treated with equal concentration of isolated EDEVs (20 µg) for 30 min prior to the addition of LPS (1 µg/ml) or vehicle. Representative traces (*panels a, b, e, f*), drop in monolayer resistance (*panels c and g*), and % permeability from the FITC monolayer flux assay (*panels d and h)* are shown. *Panels a, b, e, f:* arrows indicate addition of EDEV and LPS: ○ EDEV from LMVEC overexpressing GFP, monolayer treated with vehicle, □ EDEV from LMVEC overexpressing GFP, monolayer treated with LPS; • EDEV from LMVEC overexpressing protein of interest, monolayer treated with vehicle; ▪ EDEV from LMVEC overexpressing protein of interest, monolayer treated with LPS. Data are presented as mean ± SEM. *n* = 5–6. *p < 0.05 vs vehicle. GFP: green fluorescent protein; EDEV: endothelial-derived extracellular vesicles; LPS: lipopolysaccharide; p18^wt^: wild-type p18; ns siRNA: non-specific control siRNA; Rab4^Q67L^: dominant active Rab4; Rab4^S22N^: dominant negative Rab4.

We have previously demonstrated a role for the endosomal protein p18 in tightening the pulmonary endothelial barrier.^17^ Given the role of EDEVs in mediating barrier integrity,^6^ we sought to investigate whether p18 overexpression had an impact on the ability of extracellular vesicles to regulate pulmonary endothelial barrier function. EDEVs were isolated from LMVECs overexpressing p18^wt^, non-endosome binding p18 mutant (p18^N39^), or GFP control. Isolated EDEVs were then used to treat a naïve monolayer, using LPS as a model of endothelial barrier disruption, and permeability was assessed via ECIS (Fig. 1a–c and e–f) and FITC-dextran monolayer flux assay (Fig. 1d and h). In the absence of LPS, endothelial permeability of the naïve monolayer was unaffected following exposure to EDEVs isolated from cells overexpressing any of the p18 or GFP constructs (Fig. 1a–d(i)). In the presence of LPS and EDEVs derived from GFP-overexpressing cells, monolayer resistance was significantly disrupted with an increase in permeability (Fig. 1). Interestingly, EDEVs derived from p18^wt^-overexpressing endothelial cells were protective against LPS-induced permeability (Fig. 1a, c, and d(i)); however, EDEVs isolated from p18^N39^-overexpressing cells exerted no protective effect (Fig. 1b–d(i)). In addition, EDEVs were isolated from LMVECs where p18 expression was knocked down with the use of specific siRNA. siRNA knockdown was confirmed by Western blotting of LMVEC lysates, with p18 protein expression significantly reduced compared to the non-specific control siRNA (ns siRNA: 87.3 ± 10.1 a.u. versus p18 siRNA: 39.8 ± 5.6 a.u). EDEVs isolated from p18 knockdown LMVEC had no impact on endothelial permeability following either vehicle or LPS treatment (Fig. 1d(ii)). These data indicate that p18 overexpression results in the production of protective EDEVs and this mechanism is reliant on p18 protein expression and on the ability of p18 to bind to the endosome.

As we have previously demonstrated that overexpression of p18^wt^ cDNA in LMVEC protects against LPS-induced permeability,^17^ we next assessed whether EDEVs exert a barrier-protective effect through releasing vesicles with elevated p18 protein within. Whilst increased expression of GFP was observed in transfected endothelial cells for p18^wt^, p18^N39^, and GFP control vector, the EDEVs derived from these cells did not display increased GFP fluorescence compared to the untransfected control (Table 1a). To examine whether p18^wt^ overexpression impacted specific features of EDEVs in the presence or absence of LPS, the extracellular vesicle concentration (Table 1b) and protein concentration of the vesicles were measured as a ratio of the total number of endothelial cells (Table 1c). LPS significantly increased the concentration of EDEVs and protein concentration in EDEVs from cells overexpressing GFP and p18^N39^ but had no effect on these parameters in EDEVs isolated from endothelial cells overexpressing p18^wt^ (Table 1b and c). This corresponds to a significant increase in permeability observed following treatment of naïve LMVEC with EDEVs isolated from cells treated with LPS (vehicle: 100 ± 8.3% versus LPS: 151.4 ± 6.9%). Taken together, these data suggest that, in settings of ARDS, barrier-disruptive, protein-rich EDEVs are released. The data also show that p18^wt^-overexpressing cells produce low-protein EDEVs, which protect the pulmonary endothelium from LPS-induced vascular leak.

**Table 1.** Characterization of endothelial cells and the extracellular vesicles isolated from endothelial cells overexpressing p18 and Rab4 constructs.

a)						
Sample	Level of fluorescence (O.D._485/530 nm_)					
	Transfected cDNA					
	UT	GFP	p18^wt^	p18^N39^	Rab4^Q67L^	Rab4^S22N^
Endothelial cells	20 ± 5	1127 ± 134	1184 ± 139	1177 ± 94	1537 ± 128	1304 ± 89
EDEV	16 ± 4	19 ± 2	14 ± 1	16 ± 2	16 ± 3	19 ± 4

**b)**						
Treatment	EDEV concentration (10^8^ vesicles/ml)					
	Transfected cDNA					
	UT	GFP	p18^wt^	p18^N39^	Rab4^Q67L^	Rab4^S22N^
Vehicle	0.18 ± 0.03	0.17 ± 0.02	0.17 ± 0.01	0.18 ± 0.02	0.18 ± 0.02	0.18 ± 0.02
LPS	1.16 ± 0.11*	1.16 ± 0.10*	0.25 ± 0.03	1.16 ± 0.09*	1.12 ± 0.10*	1.04 ± 0.05*

**c)**						
Treatment	EDEV protein concentration/endothelial cell number					
	Transfected cDNA					
	UT	GFP	p18^wt^	p18^N39^	Rab4^Q67L^	Rab4^S22N^
Vehicle	N.D	0.10 ± 0.016	0.10 ± 0.016	0.14 ± 0.022	0.15 ± 0.032	0.17 ± 0.031
LPS	N.D.	0.33 ± 0.020*	0.12 ± 0.012	0.33 ± 0.093*	0.31 ± 0.039*	0.31 ± 0.036*

Notes: LMVECs were transiently transfected with p18^wt^, p18^N39^, Rab4^Q67L^, Rab4^S22N^, or GFP cDNA. Following 48 h, EDEV were isolated from the media. Table a: The level of GFP fluorescence of both endothelial cells (1 × 10^6^) and isolated EDEV (20 µg) was measured at 488 nm. Background fluorescence, using DPBS, was quantified as 15 ± 7 O.D. In a separate experiment, following 48 h from transient transfection, LMVECs were treated with LPS (1 µg/ml) for a further 6 h. Endothelial cell number was determined and EDEV were isolated from the media. Table b: The concentration of EDEV was quantified using the qNano Gold, normalized to media as a control, and is presented as 10^8^ vesicles/ml. EDEV in media (no cells present) were quantified as below accurate detection (<0.00001 × 10^8^ vesicles/ml). Table c: Protein concentration of EDEV (mg/ml) is shown as a ratio of endothelial cell number (×10^6^). Data are presented as mean ± SEM. *n* = 6.

* *p* < 0.05 vs vehicle.

UT: un-transfected control; N.D.: not done; EDEV: endothelial-derived extracellular vesicles; GFP: green fluorescent protein; p18^wt^: wild-type p18; p18^N39^: non-endosomal binding p18 mutant; Rab4^Q67L^: dominant active Rab4; Rab4^S22N^: dominant negative Rab4.

### Extracellular vesicles derived from endothelial cells overexpressing dominant active or non-endosome binding Rab4 exert no effect on LPS-induced barrier permeability

To assess whether this protective role of p18 is linked to its role in recycling cargo to the cell surface, the effect of the pro-recycling Rab4 GTPase was studied using the constitutively active Rab4^Q67L^ and dominant negative Rab4^S22N^ mutants. Previous studies have demonstrated a role for dominant active Rab4 in protection of the pulmonary endothelium whilst the non-endosomal binding Rab4 disrupts the endothelial monolayer.^18^ In the present study, EDEVs from both Rab4^Q67L^- and Rab4^S22N^-overexpressing LMVEC exerted no effect on naïve LMVEC monolayers, either in the presence or absence of LPS, similar to EDEVs from GFP-overexpressing cells (Fig. 1e–h). As for EDEVs from p18^wt^-overexpressing endothelial cells, GFP fluorescence was observed at low levels in the vesicles (Table 1a). Interestingly, extracellular vesicle concentration (Table 1b) and protein concentration in EDEVs (Table 1c) from Rab4^Q67L^- or Rab4^S22N^-overexpressing cells was significantly higher when LMVECs were exposed to LPS, similar to EDEVs from GFP-overexpressing cells. These studies thus demonstrate that the effect of p18 on pathogenicity of extracellular vesicles is independent of Rab4-positive endosomal recycling.

### Extracellular vesicles derived from endothelial cells over expressing p18^wt^ display an altered miRNA profile which mediates barrier protective effects

**Fig. 2.**
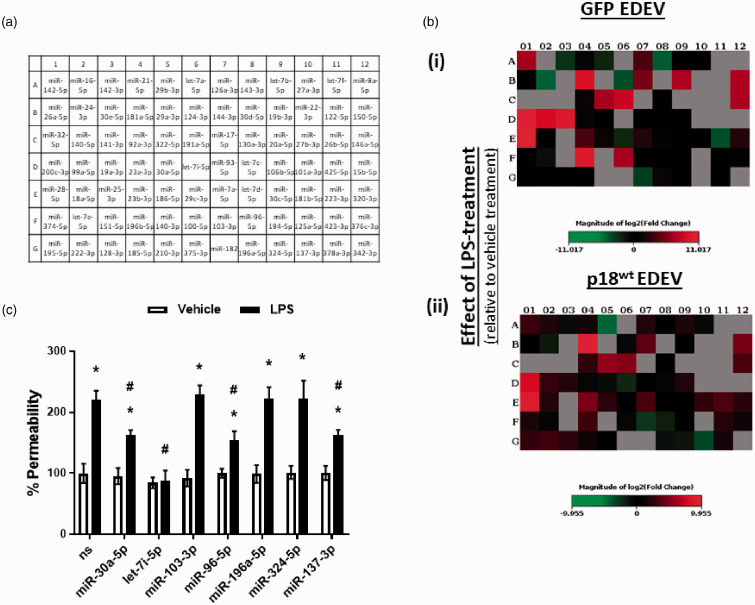
p18 overexpression regulates the release of barrier-protective miRNA in extracellular vesicles released from PAEC. Equivalent numbers of LMVECs were transiently transfected with p18^wt^ or GFP cDNA as a vector control (*panel b*) or transfected with miRNA mimics and a scrambled, non-specific (ns) control (*panel c*). Following 42 h, cells were exposed to LPS, or saline control, for a further 6 h. *Panel b:* EDEV were then collected for RNA extraction and miScript array analysis (*panel a)*. Heat maps demonstrating the change in miRNA profile were analyzed using Qiagen GeneGlobe Data Analysis center. miRNA expression profile in EDEVs from cells overexpressing GFP *(panel b(i))* or p18^wt^ *(panel b(ii))* following exposure to LPS. *Panel c:* % permeability of the endothelial monolayer measured by FITC monolayer flux assay. Data are presented as mean ± SEM. *n* = 5–6. *p < 0.05 vs vehicle for LPS, ^#^*p* < 0.05 vs ns miRNA treated with LPS. GFP: green fluorescent protein; EDEV: endothelial-derived extracellular vesicles; p18^wt^: wild-type p18; LPS: lipopolysaccharide.

We next sought to understand the potential mechanism through which EDEVs isolated from LMVEC overexpressing p18^wt^ could protect the pulmonary endothelium. A range of studies have demonstrated the key role of miRNAs in regulating endothelial barrier function^27–29^ and the potential for EDEVs to act as carriers for these miRNA.^9^ Our next experiments therefore studied the profile of miRNA present within EDEVs isolated from cells overexpressing p18^wt^ or GFP control. In untreated cells, the expression profile for miRNA in EDEVs was considerably altered in cells overexpressing p18^wt^ compared to GFP control (Table 2a). Following exposure to LPS, there were also differences in miRNA expression in EDEVs isolated from cells overexpressing p18^wt^ compared to GFP (Table 2b, Fig. 2b). Interestingly, LPS exposure causes upregulation of 9 miRNAs and downregulation of 21 miRNAs in EDEVs isolated from GFP-overexpressing cells (Table 2b). In contrast, in EDEVs isolated from cells overexpressing p18^wt^, there were 10 miRNAs upregulated and 33 miRNAs downregulated following LPS exposure (Table 2b). Of note, EDEVs isolated from p18^wt^-overexpressing cells, both treated and untreated with LPS, displayed upregulation of seven miRNAs which were unique to this construct (miR-30a-5p, let-7i-5p, miR-103-3p, miR-96-5p, miR-196a-5p, miR-324-5p, and miR-137-3p) (Table 2a (i) and b (i)). To establish whether these miRNA play a role in the barrier-protective effect of these EDEVs, LMVECs were transfected with miRNA mimics and endothelial permeability was assessed using the monolayer flux assay. To demonstrate transfection efficiency with these mimics, cel-miR67-Dy547 control was used and a significant increase in fluorescence was observed from 1351 ± 105 mean fluorescence units (MFU) compared to 369 ± 78 MFU in the scrambled, non-specific miRNA mimic control. There was, however, no significant effect of transfection of cel-miR67-Dy547 on cell viability, measured by CCK8 assay (data not shown). At baseline conditions, transfection with all miRNA mimics had no significant effect on endothelial monolayer basal permeability (Fig. 2c). Following exposure to miR-103-3p, miR-196a-5p, and miR-324-50 mimics, there was no significant change in LPS-induced endothelial monolayer permeability (Fig. 2c). Interestingly, transfection with mimics for miR-30a-5p, miR-96-5p, and miR-137-5p significantly reduced permeability induced by LPS whilst only let-7i-5p miRNA mimic was able to completely block LPS-mediated leak (Fig. 2c). These data indicate that p18^wt^ regulates the miRNA cargo of EDEVs and that these cargo include barrier-protective miRNAs in in vitro settings of ARDS.

**Table 2.** miRNA expression in EDEVs isolated from endothelial cells overexpressing p18^wt^ in the presence and absence of LPS.

a) i)					
Upregulated miRNA in p18^wt^-overexpressing cells exposed to vehicle (relative to GFP-overexpressing cells exposed to vehicle)					
miRNA	Fold upregulation	Adjusted *p* value	miRNA	Fold upregulation	Adjusted *p* value
**miR-142-5p**	**0.395**	**0.04126**	miR-19a-3p	0.864	0.46892
**miR-16-5p**	**0.180**	**0.00721**	**miR-106b-5p**	**6.770**	**0.00281**
**let-7b-5p**	**4.479**	**0.03786**	**miR-101a-3p**	**0.162**	**0.03392**
let-7f-5p	0.180	0.06390	**miR-425-5p**	**0.982**	**0.00740**
**miR-30e-5p**	**0.172**	**0.00297**	**miR-15b-5p**	**0.412**	**0.00426**
miR-181a-5p	0.876	0.14804	miR-28-5p	0.027	0.24177
**miR-29a-3p**	**0.018**	**0.00164**	**miR-18a-5p**	**1.328**	**0.00195**
miR-144-3p	0.001	0.24574	**let-7i-5p**	**0.253**	**0.00313**
**miR-30d-5p**	**6.817**	**0.00915**	**miR-222-3p**	**0.757**	**0.00284**
miR-19b-3p	0.122	0.16661	**miR-23b-3p**	**2.512**	**0.03225**
**miR-22-3p**	**0.225**	**0.00256**	**miR-186-5p**	**0.701**	**0.00491**
miR-122-5p	0.027	0.06574	**miR-7a-5p**	**2.084**	**0.00952**
miR-150-5p	0.021	0.05910	**miR-30c-5p**	**0.219**	**0.00140**
**miR-32-5p**	**2.671**	**0.02835**	**miR-125a-5p**	**0.121**	**0.00117**
**miR-140-5p**	**0.470**	**0.04396**	**miR-151-5p**	**0.458**	**0.00941**
**miR-141-3p**	**0.054**	**0.00241**	miR-196b-5p	0.453	0.36637
miR-92a-3p	0.072	0.39677	**miR-140-3p**	**0.102**	**0.00278**
**miR-322-5p**	**3.694**	**0.01813**	miR-100-5p	0.029	0.79671
miR-191a-5p	0.712	0.14738	**miR-103-3p**	**0.473**	**0.00567**
**miR-17-5p**	**5.348**	**0.01930**	**miR-96-5p**	**0.344**	**0.02990**
**miR-9a-5p**	**0.127**	**0.00132**	**miR-320-3p**	**1.239**	**0.00421**
miR-26a-5p	0.330	0.24840	**let-7a-5p**	**0.228**	**0.00327**
**miR-20a-5p**	**9.817**	**0.02835**	miR-126a-3p	0.088	0.26361
miR-27b-3p	0.023	0.07468	**miR-342-3p**	**7.009**	**0.03275**
**miR-26b-5p**	**0.312**	**0.00384**	**miR-375-3p**	**0.043**	**0.04912**
miR-146a-5p	0.483	0.06670	**miR-196a-5p**	**0.837**	**0.00323**
miR-200c-3p	1.624	0.36359	**miR-324-5p**	**0.664**	**0.04302**
**miR-30a-5p**	**7.712**	**0.01102**	**miR-137-3p**	**8.981**	**0.00166**

a) ii)					
Downregulated miRNA in p18^wt^-overexpressing cells exposed to vehicle (relative to GFP-overexpressing cells exposed to vehicle)					
miRNA	Fold downregulation	Adjusted *p* value	miRNA	Fold downregulation	Adjusted *p* value
**miR-142-3p**	**–3.110**	***0.04152***	**let-7c-5p**	**–0.141**	***0.00757***
**miR-21-5p**	**–0.501**	***0.05038***	**miR-423-3p**	**–0.150**	***0.00155***
**miR-29b-3p**	**–1.040**	***0.04332***	**miR-376c-3p**	**–1.306**	***0.00109***
**miR-143-3p**	**–9.260**	***0.01100***	miR-195-5p	–0.058	*0.08020*
**miR-24-3p**	**–0.085**	***0.05068***	**miR-25-3p**	**–0.014**	***0.00187***
miR-27a-3p	–0.007	*0.06264*	**miR-29c-3p**	**–0.131**	***0.00758***
**miR-124-3p**	**–0.181**	***0.00705***	**let-7d-5p**	**–0.306**	***0.00460***
**miR-128-3p**	**–0.204**	***0.00818***	**miR-181b-5p**	**–0.900**	***0.01916***
**miR-185-5p**	**–0.030**	***0.00210***	**miR-223-3p**	**–13.977**	***0.02146***
**miR-210-3p**	**–0.018**	***0.00387***	**miR-194-5p**	**–0.136**	***0.00715***
**miR-23a-3p**	**–0.065**	***0.05075***	**miR-374-5p**	**–0.706**	***0.00146***
miR-99a-5p	–0.050	*0.09077*	**let-7e-5p**	**–0.293**	***0.00122***
**miR-130a-3p**	**–0.101**	***0.03982***	**miR-182**	**–1.512**	***0.03445***
**miR-93-5p**	**–1.227**	***0.04400***	**miR-378a-3p**	**–4.817**	***0.02405***

b) i)						
Upregulated miRNA in cells exposed to LPS (relative to cells exposed to vehicle)						
cDNA transfected	GFP	Fold upregulation	Adjusted *p* value	p18^wt^	Fold upregulation	Adjusted *p* value
**Common miRNA**	**miR-142-3p**	**0.531**	***0.04854***	**miR-142-3p**	**0.730**	***0.04542***
	**miR-29b-3p**	**0.157**	***0.04671***	**miR-29b-3p**	**0.092**	***0.01197***
	**miR-24-3p**	**1.028**	***0.02386***	**miR-24-3p**	**0.970**	***0.01939***
**Differences in miRNA**	**miR-124-3p**	**0.885**	***0.02851***	miR-30a-5p	2.648	*0.09452*
	**miR-29c-3p**	**0.539**	***0.04350***	**let-7i-5p**	**1.315**	***0.01741***
	**miR-223-3p**	**1.915**	***0.00706***	**miR-103-3p**	**1.701**	***0.04301***
	**let-7e-5p**	**1.443**	***0.04229***	miR-96-5p	0.359	*0.06161*
	**miR-182**	**1.798**	***0.02560***	**miR-196a-5p**	**1.124**	***0.00217***
	**miR-143-3p**	**1.382**	***0.05025***	**miR-324-5p**	**1.262**	***0.00982***
				**miR-137-3p**	**1.374**	***0.01883***

b) ii)						
Downregulated miRNA in cells exposed to LPS (relative to cells exposed to vehicle)						
cDNA transfected	GFP	Fold downregulation	Adjusted *p* value	p18^wt^	Fold downregulation	Adjusted *p* value
**Common miRNA**	**miR-142-5p**	**–0.835**	***0.04831***	**miR-142-5p**	***–0.731***	***0.01006***
	**miR-16-5p**	**–1.675**	***0.0256***	**miR-16-5p**	***–1.319***	***0.01809***
	**miR-126a-3p**	**–0.909**	***0.01733***	**miR-126a-3p**	***–1.195***	***0.01229***
	**miR-181a-5p**	**–2.301**	***0.01961***	**miR-181a-5p**	***–1.791***	***0.0416***
	miR-144-3p	–0.774	*0.21246*	**miR-144-3p**	***–0.633***	***0.03298***
	**miR-150-5p**	**–0.836**	***0.03441***	miR-150-5p	*–1.031*	*0.08415*
	miR-92a-3p	–0.145	*0.09247*	miR-92a-3p	*–0.293*	*0.05467*
	**miR-322-5p**	**–0.631**	***0.02535***	miR-322-5p	*–0.515*	*0.07646*
	**miR-191a-5p**	**–1.437**	***0.01125***	**miR-191a-5p**	***–1.328***	***0.02054***
	**miR-146a-5p**	**–2.160**	***0.00921***	**miR-146a-5p**	***–2.016***	***0.01633***
	**miR-200c-3p**	**–1.905**	***0.01545***	**miR-200c-3p**	***–2.392***	***0.02501***
	**miR-99a-5p**	**–0.366**	***0.01849***	**miR-99a-5p**	***–0.527***	***0.04049***
	**miR-19a-3p**	**–2.050**	***0.00273***	**miR-19a-3p**	***–3.109***	***0.03433***
	miR-28-5p	–1.138	*0.08351*	**miR-28-5p**	***–1.583***	***0.04108***
	**miR-23b-3p**	**–0.479**	***0.02628***	miR-23b-3p	*–0.927*	*0.06066*
	**miR-186-5p**	**–2.027**	***0.03024***	**miR-186-5p**	***–2.210***	***0.01858***
	**miR-7a-5p**	**–1.729**	***0.00747***	**miR-7a-5p**	***–1.850***	***0.01917***
	**miR-320-3p**	**–0.668**	***0.01615***	miR-320-3p	*–0.886*	*0.0672*
	**miR-196b-5p**	**–0.395**	***0.00883***	**miR-196b-5p**	***–0.135***	***0.01665***
	**miR-196a-5p**	**–0.006**	***0.00867***	**miR-196a-5p**	***–0.017***	***0.03588***
**Differences in miRNA**	**miR-19b-3p**	**–0.339**	***0.01249***	**miR-21-5p**	***–1.281***	***0.02489***
	**miR-100-5p**	**–0.244**	***0.04831***	**let-7b-5p**	***–3.022***	***0.04926***
				**miR-106b-5p**	***–1.583***	***0.02775***
				**miR-18a-5p**	***–1.549***	***0.03768***
				**miR-181b-5p**	***–0.505***	***0.02321***
				**miR-223-3p**	***–0.192***	***0.01652***
				**miR-374-5p**	***–0.672***	***0.01767***
				**miR-423-3p**	***–0.925***	***0.04923***
				miR-376c-3p	*–0.382*	*0.06346*
				**miR-195-5p**	***–1.533***	***0.01962***
				miR-222-3p	*–0.672*	*0.07756*
				miR-128-3p	*–0.880*	*0.05555*
				**miR-185-5p**	***–1.292***	***0.01253***
				**miR-378a-3p**	***–1.143***	***0.01006***

Notes: LMVECs were transiently transfected with p18^wt^ or GFP cDNA. Following 48 h from transient transfection, LMVECs were treated with vehicle (a) or LPS (1 µg/ml) (b) for a further 6 h. EDEV were isolated from the media, miRNA content was assessed using the miScript system and expressed as upregulated (i) or downregulated (ii) when compared to either GFP (a) or vehicle control (vehicle for LPS) (b). In panel (b), “common miRNAs” refers to those found in EDEVs isolated from both GFP- and p18^wt^-overexpressing cells treated with LPS. “Differences in miRNA” refers to miRNA which were found only in either GFP-overexpressing cells treated with LPS or p18^wt^-overexpressing cells treated with LPS. *n* = 4, expression data expressed as fold upregulation or downregulation, adjusted *p* value using the Benjamini–Hochberg correction. miRNA and *p* values in bold denotes a statistically-significant (adjusted *p* < 0.05) relative to GFP-overexpressing cells (panel a) or vehicle-treated cells (panel b).

p18^wt^: wild-type p18; GFP; green fluorescent protein; LPS: lipopolysaccharide.

### p18 expression is downregulated in lungs and pulmonary vasculature in settings of ARDS

**Fig. 3.**
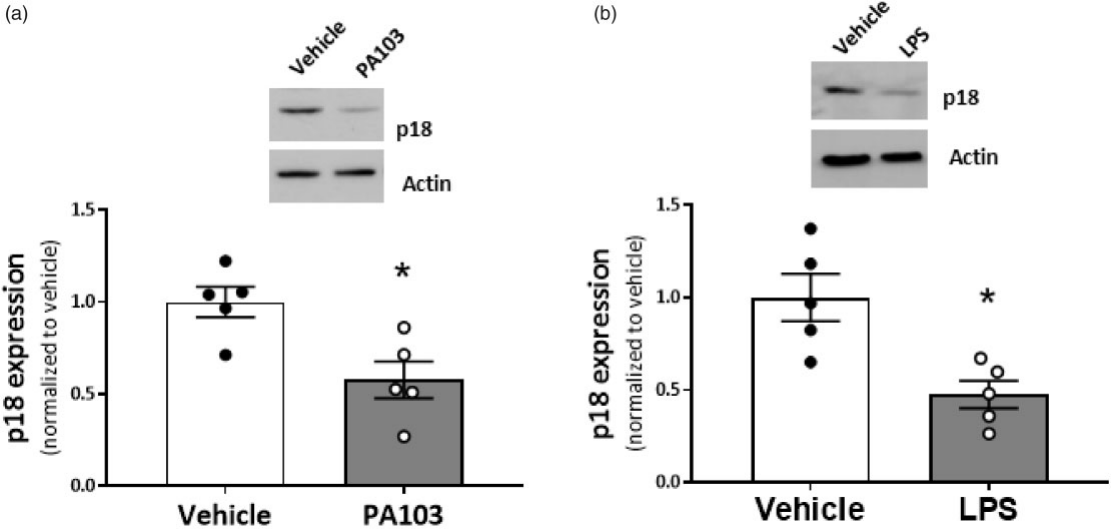
p18 expression is downregulated in settings of ARDS. (a) Lung homogenates (100 µg) from mice exposed to *Pseudomonas aeruginosa* (PA103), or PBS vehicle, for four hours were assessed by Western blotting for the expression of p18. (b) Lysates from lung microvascular endothelial cells (50 µg) exposed to LPS, or saline control, for six hours. Densitometry was performed to show protein expression, as a ratio compared to load control (actin) and normalized to vehicle control. Representative immunoblots are shown. Data are presented as mean ± SEM *n* = 5. *p < 0.05 vs vehicle. LPS: lipopolysaccharide.

Given the protective role of p18 in the pulmonary vasculature, and the link between ARDS and pathogenic EDEVs,^8^ our next experiments studied whether p18 expression in the pulmonary vasculature is affected in settings of ARDS. The *P. aeruginosa* in vivo model of pulmonary edema was used which we have previously demonstrated causes a significant 41.5% increase in wet-to-dry lung weight ratio.^17,18^ In the present study, we observed a significant decrease in p18 expression in lung homogenates from *P. aeruginosa*-infected mice, compared to saline controls (Fig. 3a). Similarly, using an in vitro model of injury, LMVECs exposed to LPS, we observed a significant decrease in p18 expression (Fig. 3b). These data demonstrate that the endosomal protein, p18, is downregulated in the pulmonary microvasculature in in vitro and in vivo settings of ARDS.

## Discussion

In the present study, we demonstrate, for the first time, that p18^wt^-overexpressing pulmonary endothelial cells release barrier-protective EDEVs. Using an established technique to isolate EDEVs, our findings show that the protein-rich EDEVs, which are shed when the endothelium is exposed to LPS, increased barrier permeability. In contrast, EDEVs from LPS-challenged cells overexpressing p18^wt^ do not display this effect on endothelial barrier integrity. We further demonstrate that this protective effect of p18 is through the ability to bind to the endosome. Findings from endothelial cells overexpressing Rab4 active and inactive mutants demonstrate that the early endosome regulator does not affect EDEVs. Our studies indicate the miRNA cargo within EDEVs released from p18^wt^-overexpressing cells, which may influence permeability of the endothelial monolayer. Finally, we demonstrate a decrease in pulmonary p18 protein expression in both in vitro and in vivo models of ARDS. Taken together, our findings indicate the importance of p18 in regulating pulmonary endothelial barrier function through the release of barrier-protective EDEVs.

Studies have demonstrated that, in settings of ARDS, the endothelium releases pathogenic EDEVs which can result in increased vascular disruption and inflammation.^8,14^ In these studies, EDEVs were isolated from the endothelium in both in vitro and in vivo experiments. Using human pulmonary arterial endothelial cells, previous studies have isolated EDEVs with the centrifugation method and demonstrated that 95% of EDEVs collected were <1 µM and double positive for annexin V and CD31.^8^ Using this same protocol, we isolated EDEVs from rat LMVECs and, similar to Letsiou et al., we observed an increase in EDEV concentration following LPS exposure.^8^ We further demonstrate that EDEVs from LPS-treated cells increased permeability of a naïve monolayer. Given the nature of the centrifugation steps, it is unlikely that remnants of the endotoxin would remain in the EDEV sample. Furthermore, endothelial injury, such as cyclic stretch, has also been shown to increase the shedding of pathogenic EDEVs from the pulmonary endothelium. Our findings therefore confirm that LPS, an in vitro model of ARDS, promotes the release of barrier-disruptive EDEVs, resulting in an increase in leak across the monolayer.

In ARDS, pathogenic EDEVs have been demonstrated to contain potentially barrier-disruptive cargo such as moesin.^8,12^ We have previously shown that p18 overexpression tightens the endothelial barrier^17^ and demonstrate that levels of p18 protein were not elevated in EDEVs from cells overexpressing the protein. These findings indicate that the specific cargo within EDEVs from p18-overexpressing cells is key to the protective effect. Extracellular vesicles can be comprised of an array of biological materials, such as miRNA, lipids, and surface proteins.^9,30,31^ Indeed in the present study, we demonstrate that p18 overexpression in cells alters the miRNA expression profile of EDEVs in an in vitro model of healthy and ARDS conditions, and specifically upregulates the expression of barrier-protective miRNA (miR-30a-5p, miR-96-5p, miR-137-5p, let-7i-5p) in EDEVs. Whilst these miRNA have not been previously associated with vascular leak, miR-137-5p and miR-30a-5p are both linked to pro-angiogenic processed whilst miR-96-5p has been shown to be downregulated in settings of oxidative stress in ARPE-19 cells.^32–34^ Interestingly, in the kidney and brain, let-7i-5p has been closely linked with anti-inflammatory processes in vitro and in vivo with elevated miRNA linked to decreased TLR4 expression and signaling.^35,36^ Thus it is possible that EDEVs rich in let-7i-5p miRNA protect the pulmonary endothelium by reducing TLR4 expression, and thus reducing the ability of LPS to cause barrier disruption. These studies indicate a potential mechanism through which p18 regulates miRNA cargo of EDEV to exert the barrier-protective effects observed. Further studies are needed, however, to assess whether these miRNAs are responsible for the protective effect exerted by EDEVs isolated from p18-overexpressing cells.

miRNAs are processed through multiple cellular steps; pre-miRNA is synthesized in the nucleus and exported, via exportin 5, into the cytoplasm for cleavage by Dicer and AGO proteins into functional miRNA.^16^ The TAR-RNA binding protein (TRBP) binds Dicer to stabilize the pre-miRNA:Dicer complex and it has been demonstrated that TRBP is regulated by mitogen activated kinase (MAPK)-extracellular signal-regulated kinase (ERK) phosphorylation.^37^ Although there have been no studies linking p18 with miRNA processing, we have previously shown that p18 activates p38-MAPK in pulmonary endothelial cells.^17^ It is therefore possible that p18 regulates miRNA processing, and thus the composition of miRNAs expressed in EDEVs, through a MAPK-dependent pathway. Further studies are therefore needed to establish the mechanism through which p18 regulates miRNA cargo in EDEVs, with the aim of identifying novel therapeutic targets to reduce endothelial permeability in settings of ARDS.

The endosome adaptor protein, p18, has been identified at the early and late endosome to regulate mTOR and MEK activity^17,20,21^; however, despite these studies, there is still limited data regarding the role of p18 in the lung. In contrast, Rab GTPases are a well-established family of endosome regulator proteins which are known to orchestrate the trafficking of endosomes through the cell. We have previously demonstrated the protective role of the endosomal proteins, Rab4 GTPase and p18, on endothelial barrier function.^17,18^ The protective effects of both proteins is mediated by the ability of Rab4 and p18 to bind to the endosome; the non-endosome binding mutants Rab4^S22N^ and p18^N39^, respectively, had no effect on endothelial permeability. However, there are two different mechanisms through which the proteins mediate endothelial barrier protection. Rab4 attenuated LPS-induced leak through suppressed ERK activity,^18^ whereas p18 enhanced trafficking of VE-cadherin to the adherens junction to attenuate LPS-induced permeability.^17^ In contrast, in the present study, EDEVs from cells overexpressing p18, but not the early endosome protein Rab4, protected the endothelium against LPS-induced permeability. These data indicate that p18-mediated release of protective EDEVs is independent of its role in trafficking the early endosome. Interestingly, p18^wt^-overexpressing endothelial cells demonstrated improved barrier function at baseline conditions,^17^ whilst the present studies demonstrate that EDEVs from p18^wt^-overexpressing cells exert no such protective effect at baseline conditions. Therefore, it is likely that p18 regulates the pulmonary endothelium through a range of different mechanisms to reduce barrier leak.

Whilst these findings indicate the importance of EDEVs on endothelial barrier function, there are several other cell types within the pulmonary system which release extracellular vesicles and have been shown to play a role in vascular function. For example, increased platelet-derived extracellular vesicles have been observed following LPS exposure to increased endothelial cell activation and disruption,^38^ whilst LPS-stimulated macrophage extracellular vesicles increase fluid and protein leak into the alveoli.^39^ Given the expression of p18 in both platelets and macrophages^40,41^ and the decrease in p18 expression in lungs from an ARDS model, further studies should be considered to assess whether this endosomal protein could also promote the release of protective extracellular vesicles from other cell types in the lungs.

## Highlights

- The endosomal protein, p18, promotes the release of protective extracellular vesicles which reduce LPS-induced leak across the pulmonary endothelial barrier.
- Extracellular vesicles released from pulmonary endothelial cells overexpressing p18^wt^ display markedly different miRNA expression profile, relative to GFP-overexpressing endothelial cells.
- p18 protein expression is downregulated in the pulmonary endothelium and lungs of in vitro and in vivo models of ARDS respectively.
